# Comprehensive review of macrophage models: primary cells and immortalized lines across species

**DOI:** 10.3389/fimmu.2025.1640935

**Published:** 2025-08-20

**Authors:** Tiansong Ding, Yuhan Du, Bin Yang, Wenfan Tian, Jiapei Li, Jinghong Xie

**Affiliations:** ^1^ Xiyuan Hospital, China Academy of Chinese Medical Sciences, Beijing, China; ^2^ Graduate School, Hebei University of Chinese Medicine, Shijiazhuang, China; ^3^ Graduate School, Beijing University of Chinese Medicine, Beijing, China

**Keywords:** macrophages, primary macrophages, immortalized macrophage cell lines, cell models, cross-species

## Abstract

In order to preserve homeostasis, macrophages—phagocytic innate immune cells—interact with different tissue types, modulating immunological responses and secreting a variety of cytokines. They are extensively dispersed throughout the body’s tissues and organs. Based on their developmental origins, tissue-resident macrophages (TRMs) in humans can be classified into those of embryonic origin and those derived from bone marrow-derived monocytes (BMDMs); embryonically derived macrophages emerge during early development, possess self-renewal capacity, and persist into adulthood in specific tissues such as microglia in the brain and Kupffer cells in the liver, whereas BMDMs originate from hematopoietic stem cells in the bone marrow via monocytic differentiation, infiltrate tissues during inflammation or injury, and differentiate into macrophages that transiently reside in tissues but lack self-renewal capability, thus requiring continuous replenishment. Because of their flexibility and diversity, macrophages participate in a variety of physiological and pathological processes by changing phenotypically and functionally in response to microenvironmental stimuli. This process is known as macrophage polarization. As a consequence, macrophage cultivation *in vitro* has emerged as a crucial biological technique for mimicking the microenvironment of different disease models. Primary macrophage models and immortalized macrophage models are two distinct types of macrophage models, each with unique origins, functions, benefits, and drawbacks. The features, advantages, disadvantages, isolation procedures, and differentiation induction techniques of primary and immortalized macrophage models are compiled in this review. It also works at the differences between various macrophage cell lines in an effort to shed light on the pathophysiology of inflammatory disorders, viral infection processes, and macrophage immunoregulatory roles.

## 1 Introduction

Elie Metchnikoff identified and named macrophages in the late 1800s (1). In addition to aiding in tissue repair, macrophages, one of the body’s primary immune cells, control the physiological and pathological aspects of immunological and inflammatory responses by presenting antigens, polarizing, and phagocytosing. Before animal studies showed that some macrophages could have evolved from red pulp progenitors in the yolk sac around 1990, it was generally accepted that all macrophages were derived from bone marrow monocytes (2, 3). These macrophages can live in adult tissues and are capable of self-renewal (4). Macrophages living in various tissues show considerable heterogeneity, depending on niche distinctions and the tissue microenvironment. The term “macrophage polarization” describes the temporary and reversible process by which macrophages acquire certain phenotypic and functional responses to signals and stimuli from the microenvironment (5). M0 macrophages differentiate into M1/M2 subtypes according to variations in signaling. Inflammatory reactions begin with M0 macrophages, which are in an inactivated condition. Pro-inflammatory M1 macrophages mainly contribute to inflammatory reactions by phagocytosing and eliminating pathogens. Anti-inflammatory M2 macrophages primarily work to reduce inflammation and encourage tissue regeneration. Immune and inflammatory responses can be modulated by controlling the M1/M2 macrophage ratio (6). The M1/M2 distinction, considered helpful for understanding macrophage functional polarization, oversimplifies the complex continuum of states *in vivo* and ignores cellular origin and tissue milieu, according to recent findings (7). For example, regulatory macrophages (Mregs) are a unique subset of macrophages that are not dependent on M1/M2 functional states. They have been found to have angiogenic and immunosuppressive properties (8) and have been effectively used in clinical trials to lessen immunosuppressive therapy and rejection after kidney transplantation (9, 10). Similarly, there are specialized populations like tumor-associated macrophages (TAMs) (11) and heme-handling macrophages (Mhem) (12). The general M1 and M2 classifications, however, continue to be helpful recommendations for examining macrophage polarization and function, improving our comprehension of macrophage functional states *in vitro*, and enabling the investigation of new treatment approaches (7).

Serious developmental disorders, such as neurodevelopmental delays, skeletal abnormalities, impaired tissue repair/remodeling, and functional disorders in the liver, spleen, reproductive system, lungs, and cardiovascular system, are facilitated by genetic abnormalities that lead to macrophage dysfunction in both humans and mice (13). Macrophages, an essential part of the human immune system, are involved in many physiological and pathological processes and are strongly linked to a number of disorders, including infections, atherosclerosis, obesity, malignancies, and asthma (14). Therefore, studying the polarization states of macrophages has become essential for treating inflammatory diseases. Thus, the development of *in vitro* cell lines through genetic manipulation, the establishment of primary cell culture models for immunological and cell biological research, and the creation of single-source immortalized cell lines with distinct backgrounds and retained functionality constitute vital strategies for tackling today’s problems.

Without going through genetic alteration or immortalization, primary cells are extracted and cultivated straight from organisms, maintaining biological activity and population features. Cell lines are stable proliferative populations created by repeatedly subculturing primordial cells. These cells are derived from malignancies or normal tissues, but they typically lose their stable chromosome composition and population specificity. Through genetic modification (e.g., introduction of SV40 large T antigen (SV40 LT), telomerase activation, or viral transformation (e.g., Epstein-Barr virus)), cells may prevent normal senescence and death during *in vitro* culture, allowing for long-term subculture and unrestricted proliferation. This process is recognized as cellular immortalization. The same is true with macrophages. Primary macrophages and immortalized murine macrophage cell lines are the two basic groups into which macrophages can be divided according to their origin. They share the following traits: (1) Primary macrophages. The limited proliferative capability, great functional heterogeneity, and closer resemblance to *in vivo* physiological states are characteristics of these cells, which are directly collected from either humans or animals. Their manipulation and culture are rather complicated, though, and they have limited stability, short survival periods, and no capacity for long-term subculture. As a result, during use, rigorous control of the experimental conditions is necessary. Furthermore, differences in the main macrophages’ source, separation, and culture methods could affect the results of experiments (15). Animals (like peritoneal macrophages [PMs] and bone marrow-derived macrophages [BMDMs]) or humans (such as peripheral blood mononuclear cells [PBMCs] and induced pluripotent stem cells [iPSCs]) can serve as primary macrophage models. (2) Immortalized macrophage cell lines. Large-scale investigations can benefit from these cell lines’ rapid growth, stability, reproducibility, independence from conditioned medium, lack of other cell types, and ease of culture and passage (16). However, immortalized macrophage cell lines are frequently created by viral infection or derived from malignant single cells/tumors, which causes genotypic and phenotypic drift throughout culture and subculture (17–19). Genetic drift compromises the reliability and reproducibility of findings, particularly in long-term studies involving multiple passages. Resultant phenotypic alterations, including those in polarization, cytokine secretion, and phagocytosis, can lead to misleading conclusions in disease modeling. Rigorous cell line authentication, use of low-passage stocks, and reporting of passage numbers are therefore essential to mitigate risks and enhance cross-study data comparability (17–19). This is in contrast to primary cells. As a consequence, macrophage cell lines may develop molecular phenotypes different from those of primary isolated cells or macrophage-specific functions. Additionally, they depart from the primary cells’ inherent functional properties by not being able to accurately mimic the intricate *in vivo* milieu. Animal-derived lines (such as RAW264.7, J774A.1, and P388D1) and human-derived lines (including THP-1, U-937, and BlaER1) are an instance of immortalized macrophage cell lines. Research on macrophages will be addressed in more detail in the sections that follow.

## 2 Primary macrophages

**Figure 1:**
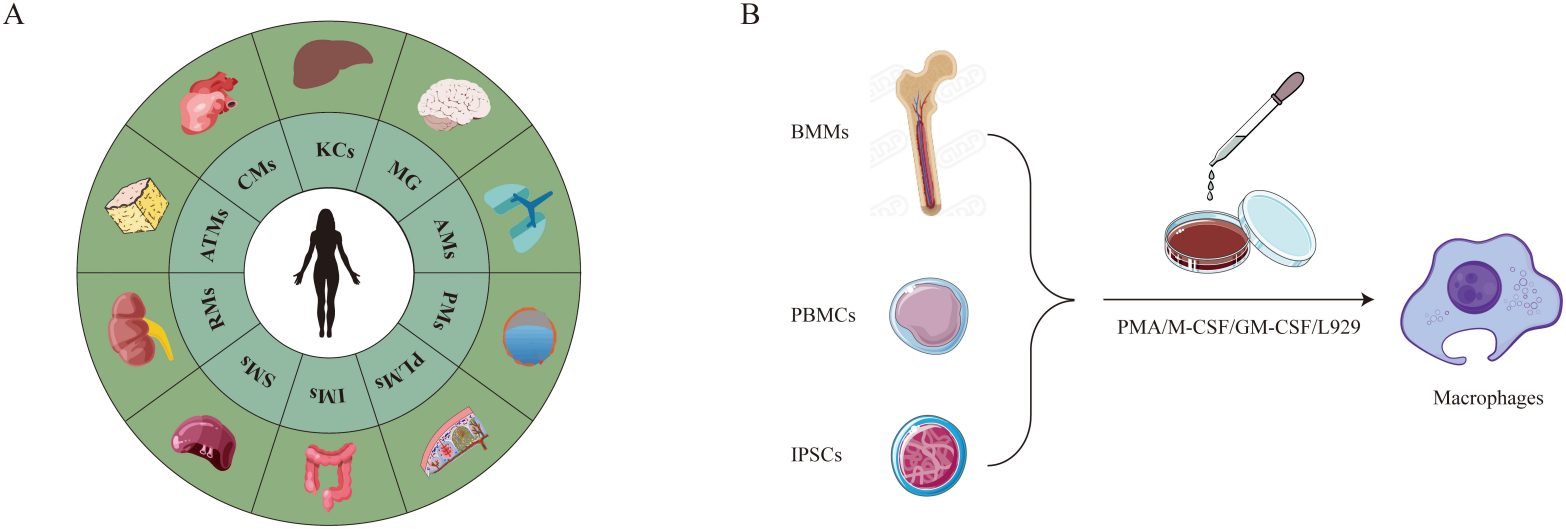
Primary macrophages. **(A)** Undergoes spontaneous differentiation without artificial induction. **(B)** Requires artificial induction of differentiation.

Initially, it was generally believed that bone marrow monocytes were the only source of macrophages (20). Around 1990, however, research on animals showed that certain macrophages could originate from the yolk sac during the development of the embryo (2, 3). These macrophages from the yolk sac can remain tissue-resident cells in adult organs and are capable of self-renewal (4). Bone marrow, peripheral blood, and tissues (e.g., as the lungs, peritoneal cavity, spleen, and placenta) are among the various sites from which primary macrophages can be isolated (**Figure 1**). Adherence selection, density gradient centrifugation, magnetic bead sorting, enzymatic digestion (collagenase/trypsin), and flow cytometry sorting are the main isolation techniques used today (21).

### 2.1 BMDMs

The primary reason for the irreplaceable core value of BMDMs is their physiological relevance. They are ideal for metabolic studies (such as glycolysis and oxidative phosphorylation assays) and validation of genetic knockout models because of their high migratory capacity, potent secretory activity (e.g., IL-1β, lysozyme), strong phagocytic capability (though they cannot trigger LC3-associated phagocytosis (LAP) (22, 23)), pronounced polarization plasticity (different M1/M2 phenotypes), and comprehensive expression of membrane proteins, including Toll-like receptors (TLRs) and scavenger receptors (24–27). The morphology of BMDMs is significantly influenced by polarization status; stimulation with LPS and IFN-γ (M1-polarizing) leads to flattened pancake-like morphology within 24 hours, whereas elongated cellular shape is promoted by IL-4 and IL-13 (M2-polarizing) (28). A Study demonstrating significantly higher numbers of aged murine BMDMs (CD11b, MHC-II, F4/80) with a roughly 2.5-fold higher percentage of M1 macrophages compared to young controls suggest that the polarization status of BMDMs may be age-dependent, while the M2 macrophage percentage did not significantly increase in aged mice (29).

#### 2.1.1 Animal-derived

In order to ensure experimental reproducibility, BMDMs closely resemble native *in vivo* macrophage characteristics and functions, thereby avoiding culture-induced variations inherent to cell lines.

BMDMs were isolated from mouse femurs and tibias using a standard bone marrow harvest and erythrocyte lysis protocol (28, 29). BMDMs differentiate from bone marrow monocytes (BMMs); however, the isolation of bone marrow monocytes is technically challenging and requires fresh preparation for each experiment. Mature BMDMs can only be obtained after a 5–7 day induction period with M-CSF or equivalent factors (e.g., secretory factors from L929 cells) in the extracellular milieu, which significantly extends experimental timelines (30, 31).

#### 2.1.2 Human-derived

Macrophages produced in human bone marrow are terminally differentiated, non-proliferative cells that resemble macrophages but are unable to passage. However, because of ethical limitations, a lack of therapeutic necessity, and logistical difficulties in procurement, their usage in research is still restricted.

### 2.2 PBMCs

Peripheral blood mononuclear cells, or PBMCs, are a mixed population that includes stem cells (0.1–0.2%), natural killer cells (5–10%), dendritic cells (1–2%), T lymphocytes (70–90%), and monocytes (10–30%) (32). They are among the primary sources of immune cells and can be further refined. Different from granulocytes and anucleate erythrocytes, peripheral blood mononuclear cells (PBMCs) have a single round or reniform nucleus and express surface markers involving MHC-II, CD14, and CD11b that facilitate antigen presentation. Yet, they do not have the myeloid lineage markers CD66b and CD15 (33, 34). The biggest leukocytes in blood, monocytes are immature when they enter circulation and are derived from bone marrow hematopoietic stem cells. They undergo directed differentiation into monocyte-derived macrophages (MDMs) when cultivated with cytokines.

#### 2.2.1 Animal-derived

PBMCs can be isolated from porcine, bovine, rabbit, or piscine sources using standardized density gradient centrifugation protocols. For bovine PBMCs isolation, representative methods involve heparinized blood collection, density-based fractionation with species-specific separation media, and adherence-based purification under controlled culture conditions (35).

#### 2.2.2 Human-derived

It is challenging to directly isolate sufficient macrophages from human tissues. Since human blood monocytes are easily obtained in high quantities and have the capacity to develop into macrophages *in vitro*, MDMs offer a great substitute. Procedure for MDMs in humans: Use density gradient centrifugation to separate PBMCs. PBMCs should be resuspended in full macrophage culture media. Adherent MDMs remain after non-adherent cells are removed.

### 2.3 Tissue-derived macrophages

#### 2.3.1 Alveolar macrophages

The pulmonary microenvironment contains at least three different types of macrophages: AMs, interstitial lung macrophages, and bronchial macrophages (36). The majority of the innate immune cells in distal lung tissues are alveolar macrophages AMs that reside on the alveolar luminal surface and have cytoplasm that is enriched with lysosomes and lamellar bodies. These cells constitutively express core surface markers such as CD206, CD169, and F4/80, and they are involved in the phagocytosis of pathogens and the efferocytosis of apoptotic cells (37, 38). AMs are a great model for researching bacterial infections since they are the first line of defense against lung pathogens. Their sources, which include humans and animals, make their isolation quite simple.

##### 2.3.1.1 Animal-derived

Bronchoalveolar lavage (BAL) is commonly used to isolate AMs from rodents (e.g., rats, mice) or mammalian species (e.g., porcine, caprine). Caprine alveolar macrophages can be isolated using standardized bronchoalveolar lavage procedures combined with density gradient centrifugation and selective adhesion protocols (39). Representative methods involve lung lavage, mononuclear cell fractionation, and adherence-based purification under controlled culture conditions. SV40 large T antigen transfection was used in a Chinese investigation (39) to accomplish caprine AMs immortalization; however, the immortalized cells showed dendritiform transformation with pseudopodial extension, which deviated morphologically from primary AMs phenotypes and compromised physiological relevance.

##### 2.3.1.2 Human-derived

The BAL fluid of patients with pulmonary diseases (such as asthma, Mycoplasma pneumoniae pneumonia, and COPD) is the primary source of human AMs, and BAL cytology is utilized as a biomarker of disease severity. AMs were isolated using a standardized clinical protocol (40) based on bronchoalveolar lavage of affected segments, processing of the lavage fluid (including centrifugation and washing), and short-term adherence culture. Cells adhering after this culture period were definitively identified as AMs by their CD68^+^ immunophenotype.

#### 2.3.2 PMs

Compared to other macrophage subtypes, PMs possess significant immunoregulatory functions, improved functional/phenotypic stability, and superior cytokine expression (41). PMs are ideal for short-term phagocytosis functional tests because they exhibit high expression of surface markers such as F4/80, CD11b, and CD14 (42, 43).

##### 2.3.2.1 Animal-derived

Direct or induced peritoneal lavage is a common method used to separate PMs from mammalian species (murine, rat, rabbit, pig, and caprine) or non-mammalian models (e.g., Oreochromis niloticus, Danio rerio).

1) Direct peritoneal lavage

PMs are commonly isolated from mice using a standard peritoneal lavage protocol (33). Following euthanasia, sterile PBS is introduced into the peritoneal cavity, the abdomen is gently manipulated, and the lavage fluid is collected. PMs are then purified by centrifugation, resuspension in culture medium, and adherence-based separation, where non-adherent cells are removed after a brief incubation period. Low yield (2–3 × 10^6^ peritoneal cells/mouse, PMs purity ~35% by F4/80^+^ flow cytometry (33)) is the main drawback. 2) Induced Peritoneal Lavage.

To create sterile inflammation and encourage PMs recruitment, inject stimulants (including thioglycollate broth (33)) into the mouse peritoneal cavity for three days in a row. Then, undertake adherent culture after obtaining these cells by peritoneal lavage. Treatment with thioglycollate enhances PMs yield by a factor of 10 per mouse (44). Even while PMs produced under inflammatory stimulation are more prevalent, the particular inflammatory conditions may cause their biological properties to differ from those of PMs in physiologically normal states. For example, thioglycollate injection-induced macrophages in mice show increased anti-inflammatory (M2) phenotypic bias (45). As a result, the choice of method should be in line with the goals and specifications of the experiment.

##### 2.3.2.2 Human-derived

Typically, human PMs are extracted from the ascites of patients suffering from serious illnesses (cirrhosis, ovarian cancer, post-dialysis uremia, etc.). Based on a study (46), there are plenty of mature, resident, and inflammatory PMs in cirrhotic ascites. Activated PMs from cirrhotic ascites release considerably higher levels of IL-6, IL-10, and TNF-α but lower levels of IL-1β and IL-12 once compared to PBMCs from healthy donors.

#### 2.3.3 Kupffer cells

KCs are tissue-resident macrophages (TRMs) that are exclusive to the liver and are identified by the surface expression of CD11b, F4/80, TIM4, and CLEC4F. These cells, which make up more than 80% of the body’s resident macrophage pool, are produced from blood monocytes that adhere to hepatic sinusoids. They support hepatic repair and regeneration and preserve inflammatory homeostasis (47). Through scavenger receptor-dependent pathways, KCs eliminate gut-derived bacterial products, senescent blood cells, apoptotic hepatocytes, and immunological complexes, reducing the severity of viral infections in the liver (48, 49). Through a variety of mechanisms, KCs are activated upon hepatic damage and are essential in a number of liver disorders. Research has shown that in mice fed a methionine/choline-deficient (MCD) diet, KCs are depleted during the early stages of non-alcoholic steatohepatitis (NASH), and that Ly6C monocyte-derived macrophages subsequently take their position as the predominant population (50). KCs have a strong capacity for self-renewal and passaging *in vitro* (51).

Primary KCs were isolated from rodent liver using established enzymatic digestion and density gradient centrifugation methods (51), with purification via selective adhesion. Cell identity was confirmed by immunophenotyping using macrophage-specific markers (CD68、CD163、CK18、CD31、α-SMA).

#### 2.3.4 Microglia

Under physiological conditions, the blood-brain barrier (BBB) restricts cellular and molecular transit into the brain, limiting macrophage infiltration. Microglia, however, serve as the resident macrophages of the central nervous system (CNS), ubiquitously distributed throughout the brain and spinal cord. They constitute 5-20% of all glial cells in the CNS, with their physiological density and function being essential for maintaining neural homeostasis (7). Beyond phagocytic capabilities, microglia play pivotal roles in CNS development and homeostasis through immune surveillance, neurogenesis support, synaptic refinement, axonal growth guidance, injury repair, and neurotrophic factor secretion (52, 53). Microglial polarization shares similarities with but exhibits greater specificity than the classical M1/M2 dichotomy: M1-polarized microglia predominantly exert pro-inflammatory and neurotoxic effects, while their M2-polarized counterparts demonstrate anti-inflammatory and neuroprotective functions. The M2 phenotype is further subclassified into three distinct subsets (M2a, M2b, and M2c), each possessing unique molecular signatures and biological functions (54, 55).

Primary microglia exhibit a complicated morphology with cytoplasmic vacuolization and short processes, and they proliferate slowly. Myeloid markers CD68, CD11b, and CD14 are first expressed by them, but with prolonged *in vitro* culture, marker expression gradually declines (56). Neonatal microglia were isolated from brain tissue through mechanical dissociation and orbital shaking separation of mixed glial cultures (57), followed by phenotypic validation with standard markers.

#### 2.3.5 Placental macrophages

A special immune system found in the placenta strikes a balance between the mother’s protection against infections and the fetus’s immunological tolerance. Decidual macrophages and Hofbauer cells are two different groups of placental macrophages (58). As demonstrated by immunohistochemical research, the placenta’s macrophage proportions fluctuate dynamically throughout pregnancy, from around 50% to 20% prior to birth (59, 60). Typically, isolation procedures include density gradient centrifugation (Ficoll or Percoll), enzymatic digestion (collagenase, DNase, and/or trypsin), and positive/negative selection employing antibodies that target CD68, CD10, or CD14 or take advantage of adhesion qualities (61). However, the translational applicability of mouse models is restricted due to the phenotypic differences between human and rat placental macrophages (62), which in turn limits the functional characterization of human placental macrophages. In view of this, the majority of research on human placental macrophages focuses on tissue section immunohistochemical analysis, with functional studies being comparatively rare (61).

#### 2.3.6 Head kidney macrophages

In teleost fish, the head kidney’s primary purpose in the early stages of life is excretion. As an adult, it develops into a vital hematological and immunological organ that functions similarly to human bone marrow and is the genesis of macrophages and phagocytic activity (63). HKMs have a distinct polarization profile from the mammalian M1/M2 paradigm. M1-polarized HKMs express CXCR3.1 and iNOS, but they do not have traditional M2 polarization; instead, they have a regulatory phenotype that is dominated by CXCR3.2 and Arg2 (64). Numerous fish species, including salmonids, Atlantic cod, and southern bluefin tuna, have well-established macrophage isolation and culture methods, which have made it possible to conduct in-depth study in fish immunology (65–68).

#### 2.3.7 Intestinal macrophages

In order to maintain intestinal microenvironmental equilibrium, IMs are essential for differentiating between dangerous microorganisms and innocuous antigens, such as dietary proteins and resident commensal flora (69). Lamina propria macrophages (LPMs) and muscularis macrophages (MMs) are two types of IMs. LPMs perform essential innate immune effector functions and act as the main sentinels against pathogens that penetrate the epithelial barrier. Living close to the enteric nervous system, MMs modulate peristalsis, protect tissue during inflammation and stress, and maintain enteric neurons by providing trophic and neuroprotective support (70, 71). With the expression of specific receptors such as Toll-like receptors (TLR3-TLR9), CD36, NOD-like receptors, and TREM2, LpMs have strong phagocytic and bactericidal action against pathogens (70). The boosted expression of tissue-protective genes (including Retnla, Mrc1, and CD163) distinguishes MMs from their LPMs in terms of transcription and morphology (70). Weigmann et al. used traditional enzymatic digestion to create an isolated procedure for mouse intestine lamina propria mononuclear cells (72). In order to isolate rat intestinal LPMs, Ana et al. improved this method using mechanically aided enzymatic dissociation, leading to increased purity and viability with less tissue input (73).

#### 2.3.8 Macrophages in other tissues

##### 2.3.8.1 Splenic macrophages

The red pulp, white pulp, and marginal zone are the three main sections of the spleen, an essential immunological organ. Red pulp macrophages (RPMs), marginal zone macrophages (MZMs), and metallophilic macrophages (MMs) are the three subsets of SMs that are distinguished by their distribution and function. RPMs phagocytose senescent erythrocytes to release heme and IL-33, promote iron recycling, and have low levels of CD169 and high levels of F4/80, CD68, and CD206 (74, 75). MZMs acquire bloodborne pathogens and control B-cell responses while not expressing F4/80 (76). Without F4/80, MMs exhibit strong CD169 expression, eliminate pathogens and apoptotic cells (76), and indirectly stimulate T lymphocytes (77). Splenic macrophages were obtained using antibody-based enrichment techniques from dissociated spleen tissue, with purity assessed by flow cytometry (78–81).

##### 2.3.8.2 Renal macrophages

Renal macrophages are necessary for immune monitoring, help maintain homeostasis, and take part in tissue damage and repair (82). These macrophages fall into two subgroups: kidney-resident macrophages and infiltrating macrophages, which are further split into Ly6C^hi^ and Ly6C^lo^ cells (83). Research shows that pro-inflammatory M1-polarized macrophages are drawn to the kidney during the early stages of ischemia/reperfusion injury (≤48 hours), where they release inflammatory mediators like TNF-α and iNOS that worsen tissue damage; genetically deleting M1 macrophages reduces renal damage (84). Effective techniques for separating murine renal macrophage populations and carrying out flow cytometry analysis are described in a work by Sarah et al. (83).

##### 2.3.8.3 Adipose tissue macrophages

ATMs have been found in humans, primates, rodents, an array of non-rodent animals, and amphibians. They appear in the stromal vascular fraction (SVF) of adipose tissue (85). Adipose tissue macrophages have the ability to either directly or indirectly contribute to the body’s energy storage (86). Although human adipose tissue can be isolated to produce primary ATMs for *in vitro* culture, mouse studies seldom use primary ATMs directly; instead, BMDMs or immortalized macrophage cell lines are used as stand-ins (87, 88). For instance, chemokines and inflammatory cues stimulate the membrane receptor CCR2, which attracts bone marrow-derived mononuclear macrophages to adipose tissue. By releasing inflammatory chemicals like TNF, these cells control the activity of adipose tissue (89, 90). However, a 3D *in vitro* system for producing and cultivating functional ATM-resident macrophages that had better metabolic rates and stronger surface marker specificity than BMDMs was described by Adele et al. (2024) (91). This 3D approach exemplifies how advanced culture systems can enhance physiological relevance compared to traditional 2D models or immortalized lines.

##### 2.3.8.4 Cardiac macrophages

CMs are the most prevalent immune cell group in the heart, making up 6–10% of all cardiac cells. They are essential for preserving cardiac homeostasis and controlling the heart’s reactions to stress (92–94). In 2007, Nahrendorf et al. published the first methodology for making single-cell suspensions from mouse hearts (95). Geetika et al.’s later improvements (96) made it possible to successfully isolate macrophages from human heart tissue. More readily available cell lines, like RAW 264.7, are frequently used in place of primary cells in experiments (97).

#### 2.3.9 iPSC-derived macrophages

Numerous techniques for producing iPSCs from somatic cells have surfaced since Yamanaka et al. converted mouse fibroblasts into iPSCs by adding the OSKM transcription factors (OCT4, SOX2, c-MYC, and KLF4) (98). Through reprogramming, human-iPSCs are created from terminally differentiated somatic cells while maintaining the ability to self-renew (99). iPSCs function and develop similarly to embryonic stem cells (ESCs); they can differentiate into any type of cell, including macrophages, and have an infinite capacity for proliferation (100). Furthermore, iPSCs synthesis does not require embryonic material, in contrast to ESCs. By making it possible to derive stable, uniform, and genetically defined macrophages from pre-engineered genotype-specific iPSCs, IPSDMs solve important issues in macrophage procurement (101). Investigating immune response mechanisms, activation/polarization dynamics, macrophage-specific functions, and pathologies linked to macrophages (such as inflammatory disorders and cancer immunotherapy) is made possible by this platform.

## 3 Immortalized macrophage cell lines

**Figure 2:**
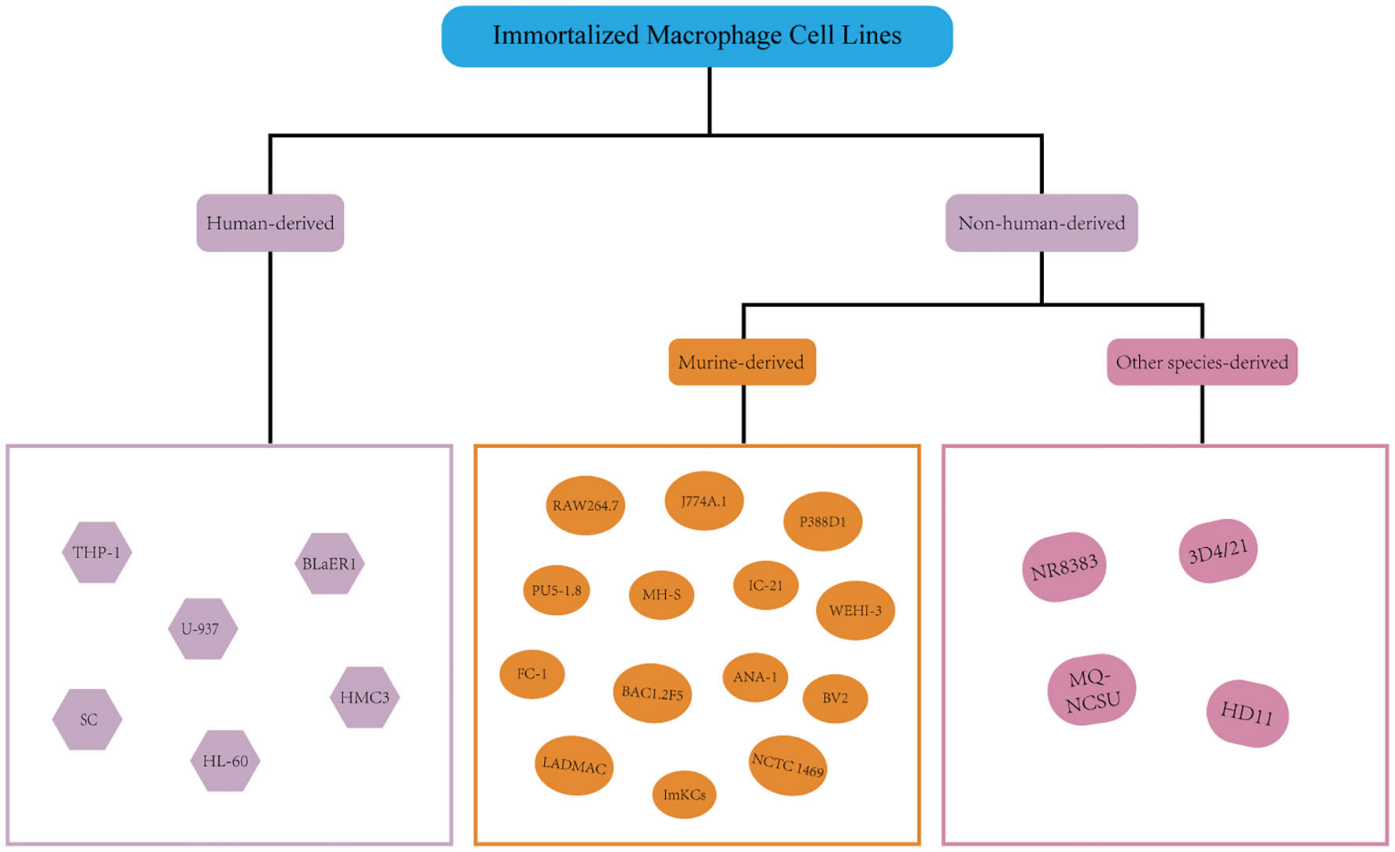
Immortalized macrophage cell lines.

Beginning in the middle to late 20th century, methods for cell immortalization were developed in order to get around the drawbacks of primary cells, such as their limited lifespan and rigorous culture needs (**Figure 2**). These strategies include activating telomerase via the TERT gene to achieve unrestricted proliferation or introducing viral oncogenes (such as the SV40 LT antigen or human papillomavirus E6/E7 proteins) (102–104).

### 3.1 Animal-derived

#### 3.1.1 RAW264.7

The RAW264.7 cell line was first established in 1976 by Raschke et al., isolated from a male BALB/c mouse injected with Abelson murine leukemia virus (MuLV) (18). These cells exhibit a round or oval morphology with dark pigmentation, strong adherence, pseudopod extension, enhanced migratory capacity, and robust phagocytic activity. Due to their capabilities in pinocytosis and phagocytosis, RAW264.7 cells are widely utilized as a macrophage model in inflammation, immunology, apoptosis, and cancer research (105). While this cell line serves as a practical tool for preliminary screening of macrophage-related factors and functions, prolonged passaging may lead to progressive depletion of macrophage-specific gene/protein expression and impaired immune functionality compared to primary macrophages or *in vivo* models. Research indicates that RAW264.7 macrophages exhibit increased expression of genes including HIF1α, ITGAL, and CD86 after 50 passages, whereas changes in ARG1, TRF2, and IRF8 expression start to occur as early as 15 passages (106).

#### 3.1.2 J774A.1

In 1968, a BALB/c mouse’s reticulum cell sarcoma gave rise to the J774A.1 cell line (17). In addition to retaining many of the functional characteristics of normal macrophages, such as phagocytosis (e.g., engulfing bacteria, apoptotic cells, or fluorescent-labeled particles), activation (inducible to M1/M2 polarization *in vitro*), and secretory activity (e.g., production of IL-1β and high levels of lysozyme), J774A.1 also demonstrates relative stability and ease of proliferation in culture (17, 107). LPS, dextran sulfate, pure protein derivative (PPD), and other substances suppress its proliferation (108). It is noteworthy that during *in vitro* culture, this cell line does not require external addition of M-CSF or other growth hormones. It is unclear, nevertheless, if J774A.1 makes M-CSF on its own to get around this requirement or if its tumor-derived origin gives it intrinsic proliferative ability that is not dependent on M-CSF signaling (109).

#### 3.1.3 P388D1

After methylcholanthrene-induced lymphoma development, DBA/2 mice were employed to create the murine monocyte/macrophage-like cell line P388D1 (110). IL-1 and lysozyme production in response to lipopolysaccharide (LPS) and phorbol myristate acetate (PMA) stimulation (111), phagocytosis of zymosan and latex microspheres (112), and surface immunoglobulin (sIg) negativity (112, 113) are among the macrophage-associated traits displayed by P388D1.

#### 3.1.4 PU5-1.8 (PU5-1R)

Originating from a spontaneous tumor in BALB/c mice, the PU5-1.8 cell line is a leukemia cell line that resembles macrophages. It has been extensively employed as an *in vitro* model for the investigation of monocyte differentiation, apoptosis, and cellular proliferation (114). PU5-1.8 cells are non-adherent and non-aggregating under conventional culture conditions (RPMI-1640 + 5% FBS + 1% penicillin/streptomycin; 5% CO_2_; 37°C), which is compatible with undifferentiated macrophage characteristics. M-CSF induction can differentiate the cells (115).

#### 3.1.5 WEHI-3

WEHI-3, first reported by Warner et al. in 1969, is a granulocytic leukemia cell line with macrophage-like properties derived from the peripheral blood of tumor-induced BALB/c mice. It exhibits sustained secretion of IL-3, lysozyme, and granulocyte colony-stimulating activity (CSA), along with phagocytic capability and expression of complement C3 receptor (C3R) (116). In intervertebral disc degeneration research, the WEHI-3 model may simulate mechanisms where M1 macrophages exacerbate inflammation through IL-1β and IL-6 secretion, while M2 macrophages promote tissue repair via TGF-β production (117). In hepatocellular carcinoma studies, WEHI-3 mimics tumor-associated macrophages (TAMs) to investigate how M2 polarization supports tumor progression through IL-1β secretion and angiogenesis promotion (118). WEHI-3 proliferates under conventional culture conditions without exogenous M-CSF, in contrast to the majority of established macrophage-like cell lines that require M-CSF for proliferation (119). It most likely depends on self-secreted IL-3 and components of the basal medium (120, 121).

#### 3.1.6 BAC1.2F5

By transforming a hybrid strain of BALB/c and A.CA mice with the SV40 virus, the murine macrophage cell line BAC1.2F5 was created (119). It retains functions including Fc-mediated phagocytosis, Ia antigen expression, IL-1 secretion, and the synthesis of lysozyme, collagenase, and esterase, but it is entirely dependent on M-CSF for survival and proliferation, mimicking the behavior of primary macrophages (122). Securioside B’s growth-inhibitory action and capacity to trigger macrophage apoptosis through the mitochondrial pathway in the presence of L-cell-conditioned media (LCCM) were examined by Satoru et al. (123) in their investigation of the drug’s effects on BAC1.2F5.

#### 3.1.7 FC-1

Yet there is limited information on FC-1. In a 1983 study, FC-1 was compared to thioglycollate-induced peritoneal macrophages in the generation of plasminogen activator and six other macrophage cell lines (WEHI-3, J774A.1, RAW264, P388D1, PU5-R, and PU5-1.8). The findings indicated that FC-1 secretes non-plasminogen-dependent proteases in addition to plasminogen activators (124).

#### 3.1.8 LADMAC

The bone marrow of BALB/c mice was utilized to create the murine monocyte/macrophage cell line LADMAC. It has poor adhesion and grows in suspension (125). The research of macrophage biology, in particular growth factor interactions and M-CSF-dependent mechanisms, has benefited greatly from the use of this cell line. In order to promote the survival and proliferation of BAC1.2F5, Wong et al. (125) showed that LADMAC cells release macrophage colony-stimulating factor (M-CSF), which can be employed in place of recombinant human CSF-1.

#### 3.1.9 IC-21

By transforming peritoneal macrophages from C57BL/6 mice with the SV40 virus, IC-21 was created (126). It maintains the phenotypic and functional characteristics of normal peritoneal macrophages, including phagocytic activity, lysozyme synthesis, particular receptors (TLR4, CD14, C3R), and antigen-presenting molecules (MHC-II). Because IC-21 is naturally adherent, it reduces experimental variability and does not require extra reagents like PMA (127). Nevertheless, IC-21 and primary peritoneal macrophages differ in the following ways: ①Enhanced erythrocyte-targeted phagocytosis is demonstrated by IC-21 (128). ②IC-21 has a more homogeneous tumor-binding capacity and binds tumor cells four times more efficiently than primary macrophages. Because of its easy cultivation and persistent, uniform tumor-binding characteristics, IC-21 is an advantageous model for understanding the molecular mechanisms behind activated macrophage subsets’ selective, high-affinity tumor contacts (129).

#### 3.1.10 NCTC 1469

In 1952, the NCTC 1469 cell line was created using the liver tissue of healthy C3H/An mice. Studies on its chemotactic activity in the 1980s showed that this cell line, which resembles macrophages, responds consistently to different chemotactic substances (such as casein) in chemotaxis tests (130). Although NCTC 1469 CB, a mutant subline of NCTC 1469, lacks phagocytic activity toward erythrocytes coated with complement and IgG, it nonetheless possesses the functional and morphological characteristics of normal macrophages (131). Since more specialized macrophage models have been established, NCTC 1469 is now mostly used in hepatic research, such as investigations on liver regeneration, lipid metabolism regulation, and antioxidant capability (132–134).

#### 3.1.11 MH-S

SV40 viral transformation of alveolar macrophages collected from male BALB/c mice aged 7 weeks resulted in the establishment of the MH-S cell line (135). This cell line maintains several characteristic traits of alveolar macrophages, such as (1) typical macrophage morphology with adhesive properties; (2) phagocytic capacity; (3) enzymatic profiles that are esterase-positive and peroxidase-negative; (4) positive expression of surface membrane antigens Mac-1 and I-A; and (5) Fc receptor-dependent phagocytosis of sheep red blood cells (SRBCs) coated with immunoglobulin G. Additionally, LPS stimulation markedly increased constitutive IL-1 production.

#### 3.1.12 ANA-1

ANA-1 is a macrophage cell line that is generated by immortalizing bone marrow cells from C57BL/6 (H-2b) mice through the J2 recombinant retrovirus, and it makes up the myeloid-monocytic lineage (136). These cells have dual adherent/suspended growth patterns, reveal morphology akin to that of macrophages, with prominent vacuolization and eccentric nuclei, and have high phagocytic activity against pathogens (e.g., Mycobacterium tuberculosis, Penicillium marneffei, and others) and particulate matter. ANA-1 expresses surface markers associated with macrophages, such as FcγR (Ly-17), Mac-1, and Ly-5, and may be involved in immune and inflammatory processes (137, 138).

#### 3.1.13 NR8383

In 1983, AMs from normal Sprague-Dawley (SD) rats collected through lung lavage served as a means to create the NR8383 cell line. NR8383 acquires an unlimited proliferative capability while maintaining normal macrophage activities through serial cloning and spontaneous immortalization (139). For *in vitro* research, especially in pulmonary inflammation, these semi-suspension cells, which have an oval or spherical shape, offer a consistent supply of highly sensitive alveolar macrophages (140). NR8383 expresses TGF-β precursor, upregulates its mRNA, and secretes pro-inflammatory cytokines (such as IL-1, TNF-β, and IL-6) when stimulated by bleomycin. The cells are extremely susceptible to endotoxins; 50% of proliferation is suppressed by 10 ng/mL LPS.

#### 3.1.14 3D4/21

In 1998, the swine alveolar macrophages that gave rise to the pig alveolar macrophage cell line 3D4/21 were immortalized through SV40 transformation. It has persistent phagocytic capacity, nonspecific esterase activity, and morphology analogous to macrophages (141). Only a small proportion of 3D4/21 cells demonstrate the capacity to phagocytose latex beads following extended incubation (3 hours to overnight), as determined by Weingartl et al. (142). These results imply that while cytokine secretion functions are unaffected by immortalization, effective phagocytosis is somewhat lost.

#### 3.1.15 HD11 and MQ-NCSU

MQ-NCSU and HD11 are immortalized cell lines of chicken macrophages. The avian myelocytomatosis virus MC29 strain has been applied to turn chicken bone marrow into the HD11 cell line, which has unmistakable macrophage-like characteristics. These include elevated generation of reactive oxygen species (ROS) and distinctive morphological characteristics (143). Derived from the spleen of chickens infected with the Marek’s disease virus, MQ-NCSU demonstrates mononuclear phagocyte lineage traits and malignant characteristics (109). According to Ahmed-Hassan et al. (144), LPS causes MQ-NCSU to undergo a type I interferon (IFN) response, which in turn causes the creation of nitric oxide (NO) and IFN-β.

#### 3.1.16 Immortalized Kupffer cells

ImKCs are isolated from hepatic tissue of male H-2Kb-tsA58 transgenic mice expressing the thermolabile mutant tsA58 of SV40 large T antigen and purified via F4/80 marker expression. These cells exhibit detectable p53 expression, elevated telomerase activity, and retained functional characteristics of primary KCs, with cytokine-induced polarization marker profiles comparable to primary counterparts. ImKCs may thus serve as functional substitutes for primary KCs in experimental systems (145).

#### 3.1.17 BV2

In 1990, E. Braschi et al. created the BV2 cell line, a commonly used immortalized murine microglial model, by transfecting primary C57BL/6 mouse microglia with v-raf/v-myc oncogenes using a retroviral vector (146). BV2 cells lack peroxidase activity but have phagocytic and nonspecific esterase activity. In addition to constitutively secreting lysozyme, they also generate TNF and IL-1 when stimulated appropriately. Using MAC3 immunoreactivity, immunohistochemical profiling validates MAC1 and MAC2 expression (146).

### 3.2 Human-derived

#### 3.2.1 THP-1

In 1980, Tsuchiya et al. developed the THP-1 cell line from the peripheral blood of a kid with acute monocytic leukemia who was barely one year old. It is frequently employed in monocyte/macrophage studies and demonstrates the capacity to differentiate into different macrophage subtypes (19). Using retinoic acid (147), vitamin D_3_ (148), or phorbol myristate acetate (PMA) (149), THP-1 cells can be separated into resting M0 macrophages based on metabolic and morphological similarities. Under certain triggers, these differentiated cells polarize into different subtypes and exhibit high expression of markers characteristic of macrophages, among them CD14 and CD68. For instance: M1 polarization: Pro-inflammatory cytokines (e.g., TNF-α and IL-6) are secreted by PMA-induced M0 macrophages stimulated with LPS and IFN-γ. M2 polarization: the production of anti-inflammatory cytokines, such as TGF-β and IL-10, is triggered by IL-4 and IL-13. However, since THP-1 lacks a defined differentiation strategy, PMA concentration must be optimized for certain experimental objectives (109).

#### 3.2.2 U-937

The pleural effusion of a male patient with generalized diffuse histiocytic lymphoma was used by Sundstrom and Nilsson in 1974 to generate the U-937 cell line (111). U-937 is regarded as a relatively young monocytic population and exhibits monocyte-like characteristics. The following factors can cause it to terminally differentiate into macrophages: M0 macrophage differentiation is induced by PMA (150). Vitamin D_3_: Encourages the development of monocytes into macrophages (151). Cytokine-driven polarization: M1 polarization is induced by IFN-γ, whereas M2 polarization is induced by IL-4 (152). U-937 allows for the functional diversity of macrophages in inflammatory and immunological responses thanks to these differentiation mechanisms.

#### 3.2.3 BLaER1

The Seraphina Burkitt lymphoma cell line was transfected to produce the human B-cell precursor leukemia cell line BLaER1. The bone marrow of a female patient with acute lymphoblastic leukemia, trisomy 8, and chromosomal rearrangement t(1;19) served as the source of its progenitor line (153). Research shows that BLaER1 cells can be effectively reprogrammed to resemble macrophages, with a transcriptome similar to that of normal macrophages and improved adhesion, phagocytosis, and quiescent characteristics (154). When compared to other monocyte models with limited editing capabilities, BLaER1 offers improved genetic manipulability owing to its compatibility with CRISPR-Cas9 gene editing in the undifferentiated B-cell state (155–157).

#### 3.2.4 SC

The only documented human monocytic cell line that emerges from healthy peripheral blood is SC, which has been used in research pertaining to macrophages (158). Using PMA induction, a Chinese research team created a model of SC-derived macrophages. Among the main conclusions are (159): Phenotypic maturation: Compared to undifferentiated SC monocytes, SC macrophages exhibited a marked elevation of CD11b and CD14. Changes in morphology: LPS treatment resulted in the production of distinct pseudopodia and an elongated spindle-shaped morphology. M1 polarization is highlighted by increased secretion of pro-inflammatory cytokines (TNF-α, IL-1β, IL-6, and IL-8) and increased expression of M1 surface markers (CD80, CD86). These findings verify that SC macrophages replicate the functional and phenotypic characteristics of typical M1-polarized macrophages.

#### 3.2.5 HL-60

A 36-year-old Caucasian woman with acute myelomonocytic leukemia gave origin to the promyelocytic cell population characterized as the HL-60 cell line (160). Using substances like PMA (161) and vitamin D_3_ (162), HL-60 cells can develop into macrophage-like cells, show phagocytic activity, and react to chemotactic stimuli. This cell line provides a special paradigm for examining how lipoprotein receptor expression and regulation are affected by macrophage development. Increased production of platelet-activating factors (PAF) is linked to HL-60 differentiation into macrophages (163). Further research showed that increased amounts of ethanolamine plasmalogens and phospholipids within arachidonic acid metabolism are correlated with macrophage-like differentiation in HL-60 (161).

#### 3.2.6 HMC3

Tardieu et al. (1995) immortalized human fetal brain-derived primary microglial cultures SV40-dependently to create the HMC3 cell line (164). Although transformed HMC3 cells retain important primary microglia physical and phenotypic characteristics, they proliferate more quickly. The astrocytic marker GFAP is negative in resting HMC3 cells, but they show significant positivity for the microglial/macrophage markers IBA1, CD68, and CD11b. When HMC3 cells are at rest, the activated microglia marker MHC-II is not visible; nevertheless, after IFN-γ treatment (10 ng/mL, 24 h), it is markedly elevated (56, 164).

## 4 Experimental applications of primary macrophages versus immortalized macrophage cell lines

### 4.1 Macrophage differentiation protocols

The study of macrophage adaptability is made possible by the capacity to induce immortalized macrophage cell lines and primary macrophages to adopt particular functional states under a range of pathophysiological circumstances. The main components of differentiation techniques include exogenous signals, endogenous signals, and particular elements or components: 1. Endogenous Signals PMA is an activator of protein kinase C (PKC) that drives monocyte-to-macrophage differentiation by activating downstream signaling cascades (e.g., NF-κB, MAPK pathways) (165). 2 Exogenous Signals: To encourage macrophage conversion, cyclosporin A causes genetic reprogramming and changes to surface receptors (166). 3 Particular Elements/Components ts (109): ① Co-culturing monocytes with macrophage-stimulating factors (typically M-CSF or GM-CSF) for five to seven days causes the monocytes to differentiate into macrophages.GM-CSF stimulates differentiation into the pro-inflammatory M1 phenotype, whereas M-CSF encourages polarization toward M2-type macrophages. Parts of Bacteria: Macrophages differentiate into M1-polarized macrophages in response to LPS. ③ Cytokines: IFN-γ stimulates M1 polarization, and when combined with LPS, it works much better. The differentiation of macrophages into M2-polarized macrophages is triggered by IL-4 and IL-13.Different cell lines have their own specific induction protocols. PMA is primarily used to induce monocytic cell lines (such as THP-1 and U937) to differentiate into macrophage-like cells. When inducing primary monocytes (e.g., PBMCs), M-CSF is typically required in combination, as PMA alone may only induce partial functional changes (such as enhanced adhesion). M-CSF serves as the main regulator for macrophage survival, proliferation, and differentiation.

Different macrophages/cell lines require different induction procedures. The main purpose of PMA is to cause monocytic cell lines (such as THP-1 and U937) to undergo differentiation into cells that resemble macrophages. Since PMA alone may only partially elicit functional changes (such as improved adhesion), M-CSF is usually needed in conjunction with PMA when producing primary monocytes (e.g., PBMCs). The primary modulator of macrophage survival, proliferation, and differentiation is M-CSF. M-CSF, which is consistently present in mouse plasma at a concentration of around 10 ng/ml, is essential for monocyte recruitment and differentiation (167). M-CSF is frequently used in *in vitro* research to encourage the differentiation of immature PBMCs and BMDMs into macrophages while preserving cell viability. These cells barely live for two to three days without M-CSF treatment (168). However, mature cell lines such as PMs, RAW264.7, and J774A.1 still need bacterial components or cytokines to induce M1/M2 polarization, even if they do not require M-CSF or other growth factors to survive *in vitro*. Macrophages are the main immune cells that react to LPS and IFN-γ in classical immunological responses. Prolonged exposure to high quantities of LPS and IFN-γ may allow the body to produce pro-inflammatory substances that can hyperactivate macrophages toward M1 polarization, escalating inflammatory responses and deteriorating disorders linked to inflammation. As a result, M1-polarizing agents for macrophages are frequently LPS and IFN-γ. On the other hand, the anti-inflammatory cytokines IL-4 and IL-13 help resolve inflammation-related illnesses, decrease inflammatory responses, and encourage macrophage M2 polarization.

The responses of THP-1 and U937 macrophage cell lines to M1/M2 polarization conditions were compared by Nascimento et al. (169). The findings demonstrated that PMA stimulation could differentiate THP-1 and U937 into M0 macrophages. While U937 demonstrated stronger responses to M2-polarizing stimuli, favoring the M2 phenotype, THP-1 demonstrated stronger responses to M1-polarizing stimuli, with a considerable tilt toward the M1 phenotype. THP-1-derived macrophages showed more phagocytic activity and ROS generation than U937-derived macrophages. Both THP-1 and U937 showed decreased phagocytic activity and increased ROS production in response to M1-polarizing stimulation. The study also showed that although M-CSF doses and treatment times were adequate to promote differentiation in primary monocytes, neither THP-1 nor U937 cells could be made to develop into macrophages by M-CSF. Similarly, after M-CSF stimulation, Aldo et al. (170) found no alterations in CD14 surface expression in THP-1 cells. Results are greatly impacted by the concentrations and lengths of treatment with induction factors, even after they are discovered. For instance, the ideal PMA concentration is still being studied, and THP-1 cells lack a reliable PMA differentiation technique (109). Systematic research on the variations in biological response among cell lines is still lacking, despite the wide range of macrophage differentiation techniques.

### 4.2 Comparison between macrophage models

In conclusion, it is critical to examine the similarities and differences among the various macrophage models that are available.

#### 4.2.1 Comparison among primary macrophages

Three primary murine macrophages—SMs, PMs, and BMDMs—were compared in terms of their properties by Zhao et al. (33). The study found that 48 hours of LPS+IFN-γ stimulation could polarize all three types of macrophages into an M1 state, while 48 hours of IL-4 stimulation could polarize them into an M2 state. While there were no appreciable variations in M2-polarizing capacity, SMs showed a greater M1-polarizing capability than the others. The most homogeneous macrophages are produced via adherent culture of BMDMs; however, their phenotype leans more toward M2. It was determined that although BMDMs offer a large number of uniform macrophages, TRMs cannot be completely replaced by them. IPSDMs showed a considerable but lesser bacterial phagocytic ability than PBMCs, according to Monkley et al. (171), while their transcriptome profiles and pro-inflammatory responses were similar. Conflicting research, however, indicates that IPSDMs tend to have an M2 phenotype, which lowers phagocytic activity (172, 173).

#### 4.2.2 Comparison among immortalized macrophage cell lines

In order to assess how J774A.1, WEHI-3, P388D1, IC-21, NCTC 1469, and U937 responded to several chemotactic agents (casein, an N-formyl tetrapeptide, and culture supernatants of murine SL2 lymphoma cells), Terheggen et al. (130) performed chemotactic activity experiments on these cells. The findings showed that the cell lines’ susceptibility to various chemoattractants varied significantly. The most potent chemotactic activity was demonstrated by J774A.1 and WEHI-3 macrophage-like cells toward casein and N-formyl tetrapeptide, respectively. Van et al. (174) examined the levels of surface antigen expression, M-CSF secretion, and peroxidase activity in five cell lines: NCTC 1469, J774A.1, WEHI-3, IC21, and P388D1. According to the study, M-CSF secretion was unique to WEHI-3 cells, whereas peroxidase activity was present in all five cell lines. M1/69 and Mac-1 antigens were significantly expressed by P388D1 cells, while they were faintly expressed by NCTC 1469 and IC21 cells. J774A.1 cells showed the reverse pattern, with strong M1/69 and low Mac-1 expression in WEHI-3 cells. Nibbering et al. (175) compared quantitative data from four cell lines (WEHI-3, P388D1, J774A.1, and PU5-1.8) on surface antigen expression (e.g., F4/80, CDb11) with data from different mononuclear phagocytes. The four macrophage-like cell lines showed clear phenotypic differences, with WEHI-3 and P388D1 exhibiting the highest association with monocytes, according to the data. None of the macrophage-like cell lines closely matched any particular mononuclear phagocyte population, even though they shared several characteristics with mature mononuclear phagocytes. In comparison to RAW 264.7 and J774A.1 cells, IC-21 cells had a higher degree of differentiation than P388D1 cells (176) and more filopodia and membrane ruffles (176, 177).

#### 4.2.3 Comparison between immortalized macrophage cell lines and primary macrophages

The phagocytic activity and polarization capacity of THP-1 and PBMCs were contrasted by Shiratori et al. (152). According to the study, THP-1 has higher phagocytic activity and a stronger propensity toward M1 polarization than PBMCs. IPSDMs exhibit more distinctive macrophage surface characteristics and express larger amounts of CD86 and MRC1 than U-937 (178). The immunobiological profile of BMDMs can only be partially replicated by THP-1 cells, according to Gaidt et al. (179). Using four macrophage cell lines (J774A.1, PU5-1.8, WEHI-3, and RAW 264.1) and newly obtained primary macrophages from C3H/He mice under standard culture conditions, Nibbering et al. (180) examined the production of N-nitrosamine during immunological stimulation *in vitro*. The findings showed that when LPS was stimulated, all cell types produced nitrite and N-nitrosomorpholine, and that IFN-γ amplified this action. According to Bodel et al. (181), J774A.1, PU5-1.8, P388D1, and WEHI-3 were still able to manufacture lysozyme and pyrogens on their own *in vitro*. Via et al. (182) assessed the ability of two human macrophage/monocyte cell lines and four murine macrophage cell lines to degrade unmodified LDL (native LDL) and acetylated low-density lipoprotein (AcLDL). According to the findings, PU5-1.8, HL-60, and U-937 are not appropriate models for researching scavenger pathways; however, P388D1, J774A.1, and RAW 264.7 are. The morphology and quantitative surface expression of C3b antibodies in primary macrophages, J774A.1, PU5-1.8, WEHI-3, and P388D1 were compared by Furth et al. (183). According to morphological data, J774A.1 and WEHI-3 cell lines were almost the same in most ways, while P388D1 was the most different from them but most comparable to primary macrophages. Furthermore, WEHI-3 and P388D1 cell lines exhibited significantly reduced surface C3b receptor expression in comparison to the other two cell lines.

Plasmid DNA, siRNA, and/or shRNA transfection has been portrayed to be possible in RAW264.7 cells, but it is very difficult in primary macrophages, where it commonly results in cellular death (184–187). Interestingly, J774.1 cells share functional similarities with primary macrophages, yet exhibit distinct responses from RAW264.7 cells: transfection with plasmid DNA induces significant cell death, whereas mRNA transfection maintains cellular viability (188). For eukaryotic cells, common transfection methods include electroporation, mechanical methods (such as gene guns), and viral vectors. Yet, since almost all known transfection processes severely impair cellular survival or obstruct their differentiation and polarization tendencies, macrophages are especially resistant to transfection techniques (188). In order to overcome these constraints, optimal transfection techniques have been suggested: Michael et al. (189) created a modified nucleofection-based method that effectively transfects THP-1 cells and allows them to differentiate into mature macrophages while maintaining cellular viability and functionality. Similarly, using an improved electroporation-based, non-viral nucleofection technique that preserved cellular integrity and physiological functioning, Maeß et al. (190) successfully transfected human THP-1 macrophages. Moreover, recent studies demonstrate that engineered nanoparticles (191) and the macrophage-specific editing (MAGE) system (192) enable highly efficient transfection of plasmid DNA/RNA in primary macrophages, surpassing conventional approaches in both delivery efficacy and specificity (**Tables 1**, **2**).

**Table 1:** Experimental applications of primary macrophages.

Cell types	Differentiation protocols	Polarization protocols	Cell markers or cytokines	Note	Source
BMDMs	① Cultivate with M-CSF (at 20 ng/ml or 100 ng/ml) for 7 days. ② Cultivate with 30% L929 for 7 days.	M1: ①IFN-γ (30 ng/ml) + LPS (100 ng/ml) ②IFN-γ (100 u/L) + LPS (100ng/ml) ③FN-γ (20 ng/ml) + LPS (100 ng/ml) M2: IL-4 (20 ng/ml) + IL-13 (20 ng/ml)	M0: CD11b, F4/80 M1: CD86, MCP-1, iNOS, IL-6, IL-1β, TNF-α, CD80, IL-12p40, MHC-II M2: CD206, Arg-1, IL-10, IL-4, CD163	BMDMs exhibit age-dependent polarization dynamics, demonstrate heightened M2 bias, and display significantly enhanced LPS responsiveness—particularly in cytokine production—compared to immortalized cell lines.	(33, 193–198)
PBMCs	Differentiated with M-CSF (20 ng/mL or 100 ng/mL) for 6 days.	M1: IFN-γ (100 u/L) + LPS (100ng/ml) M2: ①IL-4 (20ng/ml) ②IL-4 (20 ng/ml) + IL-13 (20 ng/ml)	M0: F4/80, CD11b, CD14 M1: CD86, TNF-α, IL-1β, CCr7, MHC-II M2: CD206	PBMCs exhibit superior bacterial phagocytic capacity to IPSDMs but is inferior to THP-1 cells.	(33, 152, 171, 196, 198–200)
AMs	/	M1: ①IFN-γ (20 ng/ml) + LPS (10 ng/ml) ②H1N1 virus infection for 4 days M2: IL-4 (20 ng/mL)	M0: CD11b, F4/80 M1: CD86, TNF-α, IL-6, IL-1β M2: CD206, IL-10	AMs exhibit comparable phagocytic capacity to BMDMs	(38, 201–203)
PMs	/	M1: LPS (100 ng/ml) M2: ① IL-4 (20 ng/ml) + IL-13 (20 ng/ml) ② IL-4 (20 ng/ml)	M0: CD14, F4/80, CD11b M1: CD38, CCR7, IL-1B, TNF-α, CD80, CD86, MHC-II, IL-6, IL-12p40 M2:IL-10	PMs exhibit a predominant M2-polarized phenotype within the tumor microenvironment (TME).	(42, 43, 198, 204, 205)
KCs	/	M1: ①LPS (100 ng/ml) + IFN-γ(30 ng/ml) ②IFN-Γ (20 ng/ml) M2: IL-4 (20 ng/ml) + IL-13 (20 ng/ml)	M0: F4/80, CD11b, CD68, CD14, TLR4 M1: CD86, iNOS, TNF-α, IL-6, IL-1β M2: IL-10, Arg-1, CD206	KCs demonstrate robust self-renewal and passaging capacity	(51, 145, 206–209)
MG	/	M1:①LPS (1 ug/ml) ②LPS (50 ng/ml) + IFN-Γ (20 ng/ml) M2: IL-4 (20 ng/ml)	M0: CD11b M1: CD86, TNF-α, iNOS, IL-6, IL-β M2: CD206, Arg-1, Ym-1, IL-10, TGF-β	Human-MGs demonstrate significantly enhanced phagocytic activity and LPS-induced inflammatory responses compared to murine-MGs.	(210–213)
HBCs	/	/	M0: CD68 M1: CD80, CD86 M2: CD163, CD206, CD209	HBCs adopt an M2 anti-inflammatory phenotype in normal pregnancy but shift to an M1 pro-inflammatory phenotype in pathological conditions including preeclampsia and spontaneous abortion.	(214, 215)
HKMs	/	M1: LPS (30 ug/ml) + IFN-Γ2 (350 ng/ml) M2: CiIL-4/13A (200 ng/mL) + CiIL-4/13B (200 ng/ml)	M1: IL-1β, CXCR 3.1、iNOS M2: IL-10, CXCR 3.2, Arg-2	HKMs lack classical M2 polarization, instead exhibiting a regulatory phenotype dominated by CXCR3.2 and Arg2 expression.	(64)
IMs	/	/	F4/80, CD11b, CD68, CD64, Ly6C, MHC-II, CX3CR1, Ly6G, SSC	Under physiological conditions, IMs display a predominance of M2-polarized subsets, while inflammatory stimuli shift the balance toward M1-dominated phenotypes.	(216)
SMs		M1: IFN-γ (100 u/L) + LPS (100ng/ml) M2: IL-4 (20ng/ml)	M0: F4/80, CD11b M1: CD86, TNF-α, IL-1β, MHC-II M2: CD206	SMs exhibit heightened propensity for M1 polarization compared to macrophages from other anatomical sites.	(33, 196)
RMs	/	/	CD45, Ly6G-, F4/80^hi^, CD11b	/	(83)
ATMs	/	M1: ① LPS (100 ng/mL) ②IFN-Γ (50 ng/ml) M2: IL-4 (10 ng/ml)	M0: F4/80, CD11b, CD45 M1: CD36, CD16/32、IL-6, TNF-α, MHC-II M2: MerTK, Dectin-1, CD36, CD206	Compared to BMDMs, ATMs demonstrate greater phenotypic specificity, particularly in surface marker profiles, and exhibit elevated metabolic activity.	(217)
CMs	/	/	M0: CD11b, F4/80、CD68, Mac-3 M1: CD86, MHC-II, TNF-α	Human-CMs exhibit significantly lower abundance compared to their murine counterparts.	(95, 96)
IPSDMs	M-CSF (100ng/ml)	M1: 100ng/ml LPS	M1:IL-6, TNFα, IL-1β, IL-12p70	①IPSDMs demonstrate robust but PBMCs-outperformed bacterial phagocytic capacity. ②IPSDMs exhibit elevated CD86 and MRC1 expression compared to U-937 cells, displaying phenotypic features characteristic of mature macrophages.	(171, 178)

M1-polarized macrophages typically exhibit a rounded morphology, whereas M2-polarized counterparts display elongated spindle-shaped or fusiform morphology (28, 218).

**Table 2:** Experimental applications of immortalized macrophage cell lines.

Types of cell lines	Species	Differentiation protocols	Polarization protocols	Cell markers or cytokines	Note	Source
THP-1	Human (Peripheral Blood)	Treatment with PMA (10, 20, 50, or 100 ng/mL) for 1–3 days	M1: ①LPS (20 ng/mL) + IFN-γ (20 ng/mL) ②LPS (100 ng/mL) + IFN-γ (20 ng/mL) M2: IL-4 (25 ng/ml) + IL-13 (25 ng/ml)	M0: CD14, CD68 M1: TNF-α, IL-1β, IL-6, MCP-1, iNOS, CD80 M2: Arg-1, IL-10, CD163, CD206	THP-1 demonstrate heightened responsiveness to M1-polarizing stimuli and superior phagocytic capacity compared to PBMCs.	(19, 152, 169, 195–197, 219, 220)
U-937	Human (pleural effusion)	Treatment with PMA (10 ng/mL) for 24 hours	M1: LPS(100 ng/mL)+ IFN-γ(20 ng/mL) M2: IL-4(20 ng/mL)+ IL-13(20 ng/mL)	M1: IL-6, IL-1β M2: IL-10, Arg-1	U937 are more sensitive to M2 stimulation.	(111, 169)
BLaER1	Human (bone marrow)	5-day incubation with 17β-estradiol (100 nM) + IL-3 (10 ng/mL) + M-CSF (10 ng/mL).	TLR ligands or oligonucleotides	M0:CD11b, CD45, CD14 M1:CD64, CD191, IL-6, TNFα, IL-1β M2:CD36, CD163	BLaER1 outperforms THP-1 in simulating PRR signal transduction.	(153, 221)
SC	Human (Peripheral Blood)	48h PMA (60 ng/mL) treatment.	M1: LPS (100 ng/mL)	M0: CD11b M1: CD80, CD86, IL-12p70, TNF-α, IL-1β, IL-6, IL-8 M2: IL-10	SC easily polarizes to the M1 type.	(159)
HL-60	Human (Peripheral Blood)	48h PMA (500 ng/mL) treatment.	M1: LPS (40 ng/ml)+ IFNγ (20 ng/ml) M2: IL-4 (20 ng/ml)	M0: CD14, CD11b M1: IP-10, TNF-α, NOS2, iNOS, CD86, CD80 M2: CD163, CD206	HL-60 demonstrate phagocytic activity and chemotactic responsiveness.	(165, 222)
RAW264.7	Murine (tumor)	/	M1: ①LPS ± FMN (1 μg/mL) ②LPS gradient (0–2000 ng/mL) M2: IL-4 (10 or 20 ng/mL)	M0: F4/80 M1: iNOS, CD80, CD86, TNF-α, IL-1β, IL-6 M2: CD163, CD206, IL-10, TGF-β	One of the most commonly used immortalized macrophage models	(18, 223–226)
J774A.1	Murine (reticular cell sarcoma)	/	M1: ①LPS (100 ng/ml) ②LPS (1 μg/mL)	M1: iNOS, TNF-α, IL-1β, IL-6, IL-18	① J774A.1 and WEHI-3 are largely comparable, but WEHI-3 exhibits high M1/69 and low Mac-1 expression, while J774A.1 shows the reciprocal pattern. ②J774A.1 exhibit robust phagocytosis.	(17, 111, 174, 176, 227, 228)
P388D1	Murine (lymphoma ascites)	/	/	/	① P388D1 constitutively expresses M1/69 and Mac-1. ② WEHI-3 and P388D1 exhibit the strongest transcriptomic correlation with monocytes. ③ P388D1 is phenotypically less differentiated than IC-21.	(110, 111, 174–176)
PU5-1.8/PU5-1R	Murine (lymphoma ascites)	/	/	/	The adhesion property of PU5-1.8/PU5-1R is relatively weak.	(111)
WEHI-3	Murine (peripheral blood)	/	/	/	① WEHI-3 sustains M-CSF-independent growth under standard culture. ② Primarily administered intraperitoneally/intravenously for murine leukemia modeling; seldom utilized as stand-alone *in vitro* models.	(116, 229, 230)
BAC1.2F5	Murine(spleen)	10% L929 (which produces CSF-1) for 7 days	/	/	BAC1.2F5 completely dependent on CSF-1 for survival and proliferation	(231, 232)
FC-1	Murine	/	/	/	Practical applications remain scarce, with only one pertinent publication identified in the current literature search.	(124)
LADMAC	Murine (bone marrow)	/	/	/	Low adhesiveness, capable of secreting M-CSF	(125)
IC-21	Murine (intraperitoneal)	/	M1: ① LPS (10, 25, 50, 100, and 200 ng/mL) ② IFN-γ (100 U/mL)	M1: IL-6, IL-1β, iNOS, COX-2, MMP-9, TNF, MCP-1, MIP-1a, MIP-1b, RANTES M2: PPAR-γ, CD206, CD163	The degree of differentiation is higher than that of P388D1 cells.	(126, 176, 233, 234)
NCTC 1469	Murine (liver)	/	/	/	It is now commonly used in liver-related research.	(132–134)
MH-S	Murine (alveolar)	/	M1: LPS (10 ug/ml, 100 ng/mL) M2: IL-4 (20 ng/ml)	M0: CD68 M1: TNF-α, IL-6, IL-1β, IL-18, CD80, MHC-II M2: CD206, IL-10	Retained the various characteristics of AMs.	(195, 235–237)
ANA-1	Murine (bone marrow)	/	M1: ①LPS (1 ug/ml, 1 mg/mL) ②Post-H1N1 infection M2: IL-4 (20 ng/ml) + IL-13 (20 ng/ml)	M0: F4/80, CD11b M1: CD86, MCP-1, CD80, iNOS, CD11c, IL-1β, IL-6, TNF-α M2: CD206, CD163, Arg-1, IL-10	Highly active phagocytosis	(136, 201, 238, 239)
NR8383	Rat (alveolar)	/	M1: ①LPS (20 ng/mL) + IFN-γ (20 ng/mL) ②LPS (1 μg/ml) M2: IL-4 (10 ng/ml) + IL-13 (10 ng/ml)	M1: TNF-α, IL-1β, IL-6, MCP-1, iNOS, TNF-α M2: CD206, YM-1, IRP-1, TFR	LPS inhibits proliferation of this cell line in a non-cytotoxic and reversible manner.	(220, 240, 241),
3D4/21	Pig (alveolar)	/	M1:IFN-γ (20 ng/ml) M2: IL-4 (10 ng/mL) + M-CSF(10 ng/mL)	M1: IL-6, IL-1β, TNF-α and iNOS M2: Arg-1, Mrc-1, STAT-6	① High susceptibility to porcine pathogens. ② Functional retention of primary AMs.	(242)
HD11	Chicken (marrow)	/	M1: LPS (2 μg/mL)	M1: CD86, iNOS, IL-1β, IL-6, TNF-α M2: CD206, IL-10, TGF-β1	Easy to polarize to the M1 type.	(243)
MQ-NCSU	Chicken (spleen)	/	M1: ① LPS (100 ng/ml) ②C. septicum	M1: TLR4, CD14, TLR2, IL-1β, IL-6, iNOS, IFN-Γ, MHC-II M2: IL-4, IL-10, IL-13	Displays characteristics of malignant cells and the mononuclear phagocyte system.	(244, 245)
ImKCs	Mouse (liver)	/	M1: IFN-Γ (20 ng/ml) M2: IL-4 (20 ng/ml) + IL-13 (20 ng/ml)	M0:F4/80, TLR4, CD14, CD45 M1:IRF-1, Gbp5 M2:Arg-1	Expresses signature genes of primary KCs, with cytokine-induced polarization markers comparable to those in KCs.	(145)
BV2	Mous (brain)	/	M1:LPS (100 ng/ml) + IFN-γ (20 ng/ml) M2: IL-4 (20 ng/ml)	M1:iNOS, CD86, TNF-α, IL-6, IL-1β M2:IL-10, CD163, Arg-1, CD206	Exhibits reduced TLR2 and TLR4 expression compared to primary microglia.	(246–248)
HMC3	Human (brain)	/	M1: LPS (1 ug/ml)	M1: COX2, iNOS, IL-1β, IL-6, TNF-α M2: Arg-1, IL-10	HMC3 cells exhibit strong positivity for IBA1, CD68, and CD11b, but are negative for GFAP. MHC-II is undetectable in resting HMC3 cells yet significantly upregulated upon IFN-γ activation (10 ng/mL, 24 h).	(56, 249)

FMN, Formononetin; PRRs: pattern recognition receptors; C. septicum, Clostridium septicum.

## 5 Conclusion

The development of macrophage research models has been fueled by the critical functions that macrophages play in a variety of physiological and pathological processes, such as innate immunity, tissue homeostasis, disease etiology, and tissue repair. BMDMs, MDMs, or TRMs customized for particular research goals are the most common experimental procedures used in primary macrophage models to mimic normal macrophage populations due to they accurately reflect *in vivo* features. Nevertheless, procurement and scalability issues are common to all primary macrophage types, severely restricting their experimental uses. Several immortalized monocyte/macrophage cell line-based models, including THP-1, U-937, RAW264.7, and J774A.1, have been developed to overcome these material limitations. Nevertheless, these cell lines are primarily derived from cancerous origins, which naturally limits their ability to accurately replicate normal macrophage physiology. Notably, primary models are preferred in cancer immunotherapy to retain patient-specific TAMs heterogeneity, whereas immortalized cells such as THP-1 serve better for mechanistic dissection in autoimmune disease remodeling due to their genetic tractability. When choosing models for certain experimental objectives, it is necessary to carefully weigh functional fidelity against operational practicality because these two paradigms show complementary strengths in current research. Emerging 3D *in vitro* models such as organoids and scaffold-based co-cultures offer promising avenues to overcome key limitations. These systems provide physiologically relevant microenvironments that recapitulate tissue architecture, cellular interactions, and spatial constraints essential for macrophage studies, as demonstrated in recent investigations.

## 6 Limitation

① Although macrophages are found in many tissues, the main subsets of macrophages discussed here are still quite small. Despite their being outside the purview of this analysis, emerging immortalized models (such as the canine DH82 macrophage line) still merit thorough functional profiling.
② This work acknowledges that the inherent complexity and context-dependent characteristics of macrophage populations hinder in-depth comparative investigations of phenotypic and functional variability among populations.
③ Distinct molecular fingerprints and signaling network dysregulations—mechanisms not mechanistically examined in this study—are probably the cause of phenotypic differences.
